# Structural and Functional Brain Alterations in End Stage Renal Disease Patients on Routine Hemodialysis: A Voxel-Based Morphometry and Resting State Functional Connectivity Study

**DOI:** 10.1371/journal.pone.0098346

**Published:** 2014-05-22

**Authors:** Yingwei Qiu, Xiaofei Lv, Huanhuan Su, Guihua Jiang, Cheng Li, Junzhang Tian

**Affiliations:** 1 Department of Medical Imaging, Guangdong No. 2 Provincial People's Hospital, Guangzhou, PR China; 2 Department of Medical Imaging, The First Affiliated Hospital of Gannan Medical University, Ganzhou, PR China; 3 Departments of Medical Imaging and Interventional Radiology, Cancer Center, Sun Yat-Sen University, Guangzhou, PR China; 4 Department of Renal Transplantation, Guangdong No. 2 Provincial People's Hospital, Guangzhou, PR China; Wake Forest School of Medicine, United States of America

## Abstract

**Background and Purpose:**

Cognitive impairment is a well-described phenomenon in end-stage renal disease (ESRD) patients. However, its pathogenesis remains poorly understood. The primary focus of this study was to examine structural and functional brain deficits in ESRD patients.

**Materials and Methods:**

Thirty ESRD patients on hemodialysis (without clinical neurological disease) and 30 age- and gender-matched control individuals (without renal or neurological problems) were recruited in a prospective, single-center study. High-resolution structural magnetic resonance imaging (MRI) and resting state functional MRI were performed on both groups to detect the subtle cerebral deficits in ESRD patients. Voxel-based morphometry was used to characterize gray matter deficits in ESRD patients. The impact of abnormal morphometry on the cerebral functional integrity was investigated by evaluating the alterations in resting state functional connectivity when brain regions with gray matter volume reduction were used as seed areas.

**Results:**

A significant decrease in gray matter volume was observed in ESRD patients in the bilateral medial orbito-prefrontal cortices, bilateral dorsal lateral prefrontal cortices, and the left middle temporal cortex. When brain regions with gray matter volume reduction were used as seed areas, the integration was found to be significantly decreased in ESRD patients in the fronto-cerebellum circuits and within prefrontal circuits. In addition, significantly enhanced functional connectivity was found between the prefrontal cortex and the left temporal cortex and within the prefrontal circuits.

**Conclusions:**

Our study revealed that both the structural and functional cerebral cortices were impaired in ESRD patients on routine hemodialysis.

## Introduction

End stage renal disease (ESRD) is the last stage (stage 5) of chronic kidney disease (CKD), and corresponds to complete or almost complete loss of kidney function. Cognitive impairment is highly prevalent in ESRD patients [1], [2]. Deficits involve a range of cognitive domains, including concentration, memory and planning, which may be associated with increased staff time in caring for the patients, greater utilization of healthcare resources, more frequent hospitalizations and an increased number of days spent in the hospital [3], [4]. Recognizing the cerebral deficits of ESRD could help us understand the underlying neuronal mechanisms and lead to earlier interventions that might reduce morbidity.

Imaging plays an important role in detecting structural and functional abnormalities of the brain in ESRD patients. Conventional MR and computed tomography (CT) imaging studies with visual assessment and manual measurements of structures of interest have demonstrated that patients with ESRD have reduced brain volumes, reduced deep white matter volumes, a high prevalence of subcortical white matter lesions, and a high incidence rate for stroke [5]–[8]. MR spectroscopy (MRS) studies have demonstrated that CKD patients (stage 4–5) without clinical signs of uremic encephalopathy showed metabolic disturbances in multiple brain regions, including the parieto-occipital white matter, the occipital grey matter, the basal ganglia and the pons [8]–[10]. Positron emission tomography (PET) studies have displayed that hemispheric oxygen [11] and glucose metabolism [12], especially for bilateral pre-frontal cortices (PFC), are depressed in patients with ESRD. However, due to the methodological limitations, insensitivity to the early and small lesions (conventional MRI and CT with visual assessment and manual measurements of structures of interest) [13], need for a predetermined region of interest, time-consuming manual measurements or subjective visual assessments (conventional MRI and CT with visual assessment and manual measurements of structures of interest and single-voxel MRS) [13], [14], and radiation exposure and low spatial resolution (PET) [15], their applications are restricted in a large cohort.

Resting state functional MRI (rs-fMRI) has the ability to record spontaneous brain activity fluctuations when subjects lie still in the scanner. Low-frequency (0.01–0.8 Hz) fluctuations of the blood-oxygen-level-dependent (BOLD) signal in the resting state are considered to be physiologically meaningful and related to spontaneous neural activity [16]. Recently, using rs-fMRI and the regional homogeneity analysis method, Liang et al. found that patients with ESRD showed decreased regional homogeneity in multiple areas of the bilateral frontal, parietal and temporal lobes. Moreover, they found the progressively decreased regional homogeneity in the default mode network (DMN), indicating that frontal and parietal lobes might be trait-related in ESRD patients with minimal nephro-encephalopathy [17]. Voxel-based morphometry (VBM) is a spatially specific and unbiased method of analyzing MR images reflecting the regional gray matter volume at a voxel scale [18]. This technique has already been successfully applied to normal aging [18], schizophrenia [19], dementia [20], mild cognitive impairment (MCI) [21], drug addicts [22] and hepatic encephalopathy [23]. In ESRD patients, Zhang and colleagues found diffusely decreased gray matter volume that was further decreased in the presence of encephalopathy [24]; while Prohovnik and coworkers found significant cerebral atrophy, most notably bilaterally in the caudate nuclei in ESRD patients [25]. These morphometric deficits may also relate to the functional integrity alterations in the ESRD patients. However, no studies have investigated the effects of the observed gray matter impairment on functional integrity. Studies combining VBM with rs-fMRI can explore the structural and functional cerebral deficits simultaneously [26], [27]. This method can be an ideal way to explore the neurobiological mechanisms of ESRD patients.

The purposes of the present study were to 1) identify brain regions with gray matter volume deficits, using voxel-based morphometry, and 2) investigate the brain network effect of these anatomic deficits in ESRD patients using the observed structural deficits as seed regions in functional connectivity analysis.

## Materials and Methods

### Participants

This prospective study was approved by the Research Ethics Review Board of the Institute of Mental Health at the Guangdong No. 2 Provincial People's Hospital. Written informed consent was obtained from all subjects. Sixty subjects, including 30 control subjects and 30 ESRD patients participated in this study. The ESRD patients were recruited from the Renal and Hemodialysis Clinics and Department of Renal Transplantation at Guangdong No. 2 Provincial People's Hospital. Demographic characteristics and chronic health conditions of each ESRD patient were obtained from the patient's electronic medical records. Laboratory values from ESRD patients included serum calcium, serum phosphorus, serum uric acid and creatinine. As part of the routine clinical care, these laboratory tests were drawn monthly on dialysis days prior to the treatment. Values for serum calcium, serum phosphorus, serum uric acid and serum urea were calculated by averaging the monthly laboratory tests for 3 consecutive months prior to MR imaging. All tests were performed at a single central laboratory using standard methods. Measured blood pressure was determined by averaging the 3 office blood pressure readings prior to MR imaging. The control group was recruited from the local community.

Exclusion criteria for both groups were as follows: a history of stroke or dementia either reported or documented in the medical chart, a history of Parkinson's or neurodegenerative disease, diabetes, alcoholism, drug abuse, psychiatric disorder, or major neurologic disorders (severe head injury, stroke, epilepsy, or visible lesions), liver function enzymes (AST and ALT) more than twice the upper limit of normal, or a hemoglobin level <10 g. In all of the ESRD patients MRI was performed on non-dialysis days to limit the effect of the potential temporal relationship between brain changes and time since last dialysis.

### MR imaging

MR data were obtained on a Philips Achieva 1.5 T Nova dual MR scanner using a 16-channel Neuro-Vascular (NV) coil. None of the subjects were taking any medications at the time of the scans. Tight but comfortable foam padding was used to minimize head motion, and earplugs were used to reduce scanner noise. Sagittal structural images (160 sagittal slices, TR = 25 ms, TE = 4.1 ms, thickness = 1.0 mm, no gap, in-plane resolution = 231×232, FOV = 230×230 mm^2^, flip angle = 30°) were acquired using a fast field echo (FFE) three-dimensional T1 weighted sequence. Resting-state functional MRI (fMRI) scans were performed by an echo planar imaging (EPI) sequence with scan parameters of TR = 3000 ms, TE = 50 ms, flip angle = 90°, matrix = 64×64, FOV = 230×230 mm^2^, slice thickness = 4.5 mm and slice gap = 0 mm. Each brain volume comprised 33 axial slices and each functional run contained 160 volumes (8 minutes). During resting state fMRI scanning, subjects were instructed to close their eyes and keep as still as possible, and not to think of anything systematically or fall asleep.

After the scan, all the participants were asked the following questions to verify the degree of their cooperation: “what were you thinking during the scan?”, “did you fall asleep just now?”, “were your eyes closed during the scan?” and “did you feel uncomfortable during the scan?” Only when the participant answered “nothing”, “no, I did not”, “yes, I kept my eyes closed” and “no, I did not feel any uncomfortable”, were their data used in the present study.

### Voxel-Based Morphometry Analysis

Structural image processing was conducted using the Voxel-based morphometry toolbox (VBM8) (http://dbm.neuro.uni-jena.de/vbm/) implemented in Statistical Parametric Mapping-8 (SPM8) (http://www.fil.ion.ucl.ac.uk/spm, Welcome Department of Imaging Neuroscience, London). VBM8 in SPM8 combines tissue segmentation, bias correction, and spatial normalization into a unified model [27]. Hidden Markov Random Fields were applied to improve accuracy of tissue segmentation (medium HMRF 0.3). Otherwise, default parameters were used. Individual brains were normalized to tissue probability maps provided by International Consortium for Brain Mapping (ICBM). The optimally processed images were smoothed with an isotropic Gaussian kernel (full-width half maximum = 12 mm). At the second level, whole brain data were modeled across the groups using analysis of covariance (ANCOVA) with total gray matter volume and age as covariates. The effects of total gray matter volume were removed to allow inferences between regional differences in gray matter volume. An absolute threshold mask of .1 was used. The significance of group differences in each region was estimated by distributional approximations from the theory of random Gaussian fields, and significance levels were set at p<0.05 (corrected for multiple comparisons). To identify the association between structural abnormalities and clinical severity of kidney disease and times of hemodialysis, the average gray matter volume values for all voxels in the abnormal areas, revealed by voxel-based morphometry, were extracted and correlated with the duration of chronic kidney disease, duration of hemodialysis and the laboratory values (serum calcium level, serum phosphorus level, serum uric acid level and serum urea values) in individual ESRD patient.

### Functional Connectivity Analysis

Preprocessing and statistical analysis of functional images were conducted using SPM8. For each subject, the first ten time points were discarded to avoid transient signal changes before magnetization reached steady-state and to allow subjects to get used to the fMRI scanning noise. Then echo-planar images were slice-time corrected and realigned to the first image in the first series and were subsequently unwarped to correct for susceptibility-by-movement interaction, subjects with head motion exceeding 1.0 mm of maximal translation (in any direction of x, y or z) or 1.0° of maximal rotation through the resting-state run were excluded from further analysis. All realigned images were spatially normalized to the Montreal Neurological Institute (MNI) echo-planar imaging template in SPM8, and each voxel was resampled to 3×3×3 mm^3^. Functional connectivity was examined using a method based on a seed voxel correlation approach [27], [28]. Since voxel-based morphometry analysis showed anatomic deficits in the bilateral medial PFC, the bilateral dorsal lateral PFC (dlPFC) and the left middle temporal gyrus, areas with gray matter volume reduction were defined as seeds for functional connectivity analysis. A reference time series for each seed was obtained by averaging the fMRI time series for all voxels within the region with anatomic deficits. Next, each time series was temporally bandpass filtered (0.01–0.08 Hz). Correlation analysis was conducted between the seed reference and the rest of the whole brain in a voxel-wise manner using the realigned images. To combine results across subjects and compute statistical significance, correlation coefficients were converted to a normal distribution by Fisher's z transform [29], [30].

For each group, individual z value maps were analyzed with a random effect one-sample t test to identify voxels showing a significant positive or negative correlation to the seed time series, with correlations thresholded using a family-wise error correction at p<0.05. For between-group comparison, two-sample t tests were used to compare z value maps between ESRD patients and matched controls, with the significance threshold of group differences set at p<0.05 using AlphaSim correction in the REST software (http://www.restfmri.net), which applied Monte Carlo simulation to calculated the probability of false positive detection by taking both the individual voxel probability thresholding and cluster size into consideration [31]. To identify the association between functional connectivity and clinical severity of kidney disease in ESRD patients, the z value of the regions that showed aberrant functional connectivity with the anatomic abnormalities (revealed by group comparison) were extracted and correlated with the duration of chronic kidney disease, duration of hemodialysis and the laboratory values (serum calcium, serum phosphorus, serum uric acid and serum urea values) in individual ESRD patient.

A complementary analysis was carried out to investigate the link between the structural and functional results, i.e., we wanted to address whether the effects observed on functional connectivity in ESRD patients could be explained by the reduced gray matter volume observed in the seed areas. Therefor, we replicated the four between-groups comparisons of the functional connectivity maps in using the gray matter volume of the respective seed areas as covariates.

## Results

### Demographic Results

Table 1 demonstrates the basic characteristics of ESRD patients and controls. There were no significant differences in age (p = 0.737), education (p = 0.506), sex composition (p = 0.559) between the ESRD and control groups. Table 2 demonstrates systolic blood pressure, diastolic blood pressure and hematocrit at the start and end of hemodialysis treatment session. In these patients, ESRD was secondary to glomerulonephritis.

**Table 1:** Demographic and Clinical Characteristics for End Stage Renal Disease (ESRD) Patients and Controls.

*Characteristic*	*Group*				
	ESRD Patients (N = 30)		Controls (N = 30)		
	Mean	SD	Mean	SD	P
Age (years)	38.8	9.6	38.1	7.0	0.737
Education (years)	9.8	5.1	10.6	4.5	0.506
Laboratory tests					
S. uric acid (mg/dl)	5.98	1.20	N/A	N/A	-
S. urea (mmol/l)	19.2	5.4	N/A	N/A	-
S. calcium (mmol/l)	2.32	0.25	N/A	N/A	-
S. Phosphorus (mmol/l)	1.30	0.24	N/A	N/A	-
Illness duration (years)	12.5	4.8	N/A	N/A	-
Dialysis duration (mouths)	17.2	6.8	N/A	N/A	-
Mean hemodialysis duration (h/w)	14.5	3.5	N/A	N/A	-
	N	%	N	%	P
Gender					
Female	7	23.3	9	30	0.559
Male	23	76.7	21	70	0.559

There were no significant differences in age (p = 0.737), education (p = 0.506), sex composition (p = 0.559) between ESRD patients and controls.

Note. N/A = not applicable.

**Table 2:** Blood pressure systolic, Blood pressure diastolic and Hematocrit of the ESRD patients at the start and end of hemodialysis treatment session.

*variable*	*Pre*	*Post*	*p*
Blood pressure systolic (mm Hg)	143±21	138±19	0.67
Blood pressure diastolic (mm Hg)	81±8	83±7	0.52
Hematocrit (vol%)	36.1±3.12	36.7±3.75	0.46

### Morphometry Analysis

**Figure 1:**
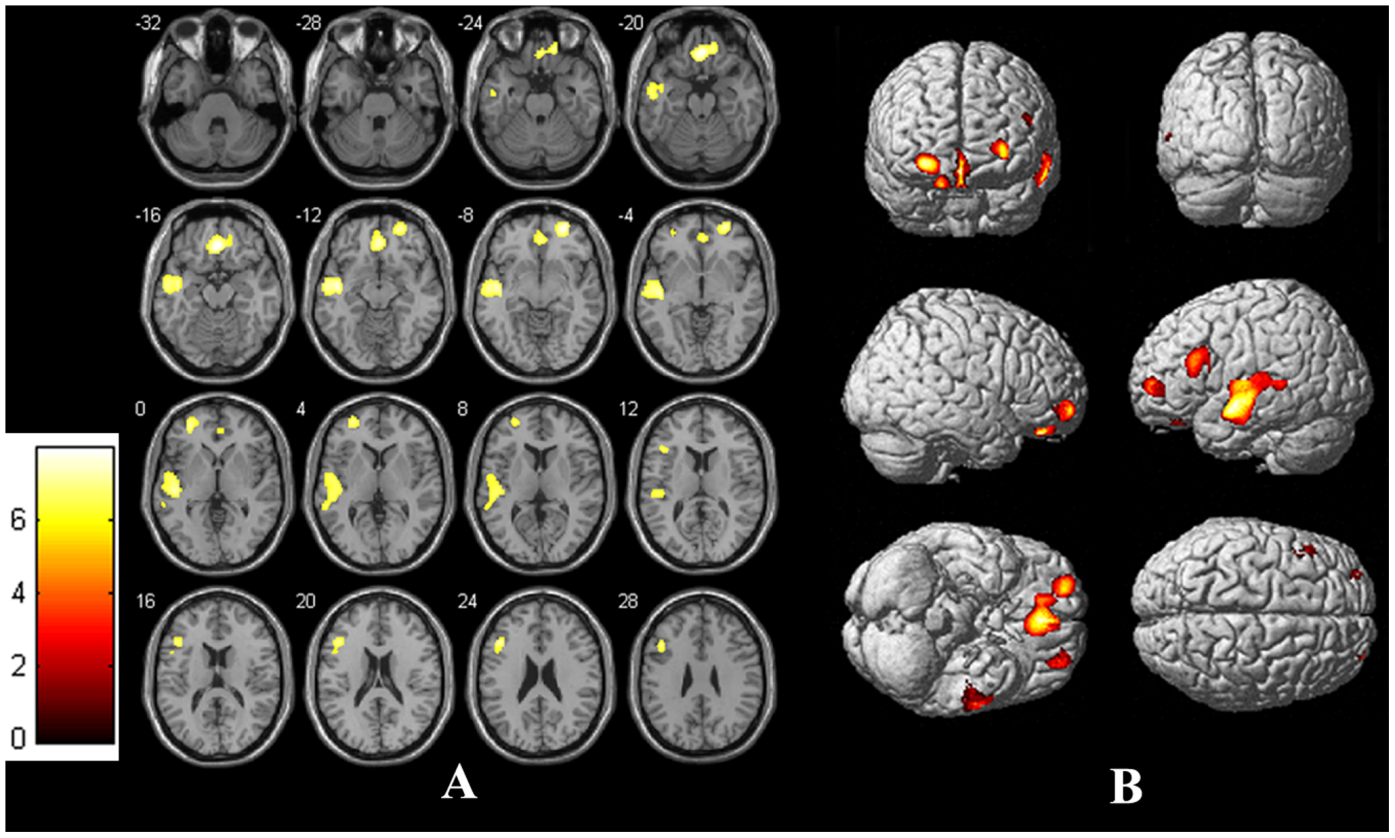
Statistical Parametric Images of Voxel-Based Morphometry Analysis for ESRD Patients and controls (Panels A: slices view, Panels B: whole brain rendering). Relative to controls, ERSD patients had significantly reduced gray matter volume in the bilateral medial orbitofrontal cortex, bilateral dorsal lateral prefrontal cortex and right middle temporal gyrus.

Relative to controls, ESRD patients showed significantly decreased gray matter volume in bilateral medial orbito-prefrontal cortex (OFC, Brodmann's area 10, 11, 32, Talairach coordinates: 1.5, 43.5, −13.5; voxel size = 328 mm^3^), left middle temporal gyrus (Brodmann's area 10, 11, 32, Talairach coordinates: −55.5, −9, −12; voxel size = 533 mm^3^), left dorsal lateral prefrontal cortex (dlPFC, Brodmann's area 10, Talairach coordinates: −30, 51, 0; voxel size = 340 mm^3^) and right dlPFC (Brodmann's area 11, Talairach coordinates: 27, 54, −9; voxel size = 182 mm^3^) (Figure 1). No significant increases in gray matter volume were found in ESRD patients compared to controls. No significant negative/positive correlations were found between the abnormal gray matter volume and the duration of chronic kidney disease, duration of hemodialysis and laboratory values for serum calcium level, phosphorus, uric acid and urea levels in ESRD patients.

### Functional Connectivity Analysis

**Figure 2:**
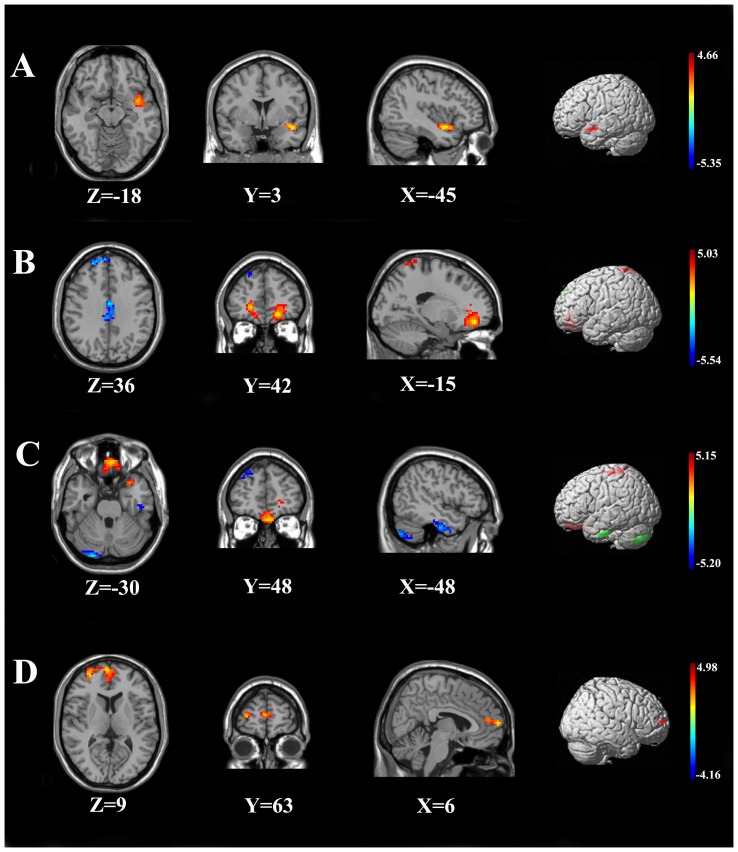
Statistical Parametric Images of Between-Group Functional Connectivity Analysis for ESRD Patients and Controls. Panels A demonstrated enhanced functional connectivity in superior temporal gyrus (Red) in ESRD patients when the seed areas were located in the left dorsal lateral prefrontal cortex ([Brodmann's area 10], panels B demonstrated reduced functional connectivity in bilateral cerebellum posterior lobe, left inferior temporal gyrus, right dlPFC (Blue), and enhanced functional connectivity in bilateral OFC, left posterior gyrus (Red) in ESRD patients when the seed areas were located in the right dorsal lateral prefrontal cortex ([Brodmann's area 11], panels C demonstrated reduced functional connectivity in bilateral cerebellum posterior lobe, right dlPFC, bilateral ACC (Blue), enhanced FC in bilateral OFC, bilateral superior parietal lobe (Red) when the seed areas were located in the medial orbito-frontal cortex ([Brodmann's area 10, 11, 32], and panels D demonstrated enhanced functional connectivity in right medial PFC (Red) in ESRD patients when the seed areas were located in the left middle temporal gyrus [Brodmann's area 10, 11, 32].

The four seed areas, where reduced gray matter volume was detected among ESRD patients, were selected for functional connectivity analysis. When the seed was located in the bilateral medial OFC, the ESRD patients showed reduced functional connectivity in the bilateral posterior cerebellar lobes, right dlPFC, bilateral ACC, and enhanced FC in bilateral OFC, bilateral superior parietal lobe than controls (Table 3, Figure 2). When the seed was located in the left dlPFC, the ESRD patients demonstrated enhanced functional connectivity in the superior temporal gyrus compared to controls (Table 3, Figure 2). When the seed was located in the right dlPFC, the ESRD patients demonstrated reduced FC in bilateral posterior cerebellar lobes, the left inferior temporal gyrus, the right dlPFC, and enhanced FC in bilateral OFC, and the left posterior gyrus (Table 3, Figure 2). When the seed was located in the left middle temporal gyrus, enhanced FC was found in the right medial PFC in ESRD patients when compared to controls (Table 3, Figure 2).

**Table 3:** Significant differences between ESRD Patients and Controls in resting state functional connectivity for the four seed areas (showed different in group comparison) and the rest of the brain.

*Seed area*	*Cluster anatomical locations (Brodmann Area)*	*Cluster size (voxel)*	*Primary peak location*	*t-score*	*ESRD n = 30 Mean (zFC)*	*Controls n = 30 Mean (zFC)*
**ESRD<Controls**						
Bilateral medial OFC	Right cerebellum posterior lobe	388	36, −84, −33	−5.5361	−0.28	−0.04
	Left cerebellum posterior lobe	258	−36, −78, −45	−4.059	−0.28	−0.03
	right dlPFC (8,9,10)	139	24,51,36	−4.371	−0.13	0.08
	bilateral ACC(23,24,31)	234	18, −39,36	−4.6875	−0.10	0.08
Right DLPFC	Left Cerebellum Posterior Lobe	92	−48, −72, −48	−4.1573	−0.15	0.05
	Right Cerebellum Posterior Lobe	89	24, −93, −30	−5.196	−0.19	0.01
	Left Inferior Temporal Gyrus (20)	120	−48, −15, −36	−4.625	−0.14	0.07
	right dlPFC	110	42,33,42	−3.749	−0.11	0.10
**ESRD>Controls**						
Bilateral medial OFC	Left OFC (10, 11, 32)	276	−21,42, −15	5.0312	0.49	0.21
	Right OFC (10, 11)	153	21,45, −3	4.8567	0.56	0.25
	Left superior parietal lobe (4,5,7)	127	−12, −45,75	4.0437	0.14	−0.08
	Right superior parietal lobe (7)	108	24, −81,51	4.7056	0.11	−0.12
Left DLPFC	Left Superior Temporal Gyrus (38)	100	−45,3, −18	4.6605	0.09	−0.15
Right DLPFC	Bilateral OFC (10,11,25,38,47)	366	0,48, −30	4.5486	0.23	0.01
	Bilateral postcentral gyrus (1,2,3,4,5,6)	110	−36, −39,66	4.8554	0.13	−0.07
Left middle temporal gyurs	Right medial PFC (10,32)	129	6 63 9	4.2882	0.12	−0.09

All the coordinates are donated by Montreal Neurological Institute (MNI) space coordinates. t-score donates the statistic value of two sample t-test by contrasting ESRD patients to controls at p<0.05 AlphaSim corrected.

The replication of the group comparisons for the four functional connectivity maps with the corresponding gray matter volume of the seeds as covariates resulted in similar results, except for the network corresponding to the seed of left dorsal lateral prefrontal cortex, which showed no significant differences between the two groups.

No significant positive or negative correlation was found between any of the Z values and the duration of chronic kidney disease, duration of hemodialysis and laboratory values for serum calcium, serum phosphorus, serum uric acid and serum serum urea values in ESRD patients.

## Discussion

Our study revealed the following important findings. First, ESRD patients have several areas of decreased gray matter volume (including the bilateral medial OFC, the bilateral dlPFC and the left middle temporal gyrus) compared with healthy controls. Second, the decrease in gray matter volume in these regions was related to the functional network integrity deficits in ESRD patients. To the best of our knowledge, this is the first systemic investigation of anatomic and functional deficits in ESRD patients on routine hemodialysis with VBM and functional connectivity methods.

The loss of gray matter volumes includes the bilateral dlPFC, the bilateral medial OFC, and the left middle temporal gyrus. The reduced gray matter volume of the prefrontal cortex in ESRD patients observed in the present study is supported by a recent histological study by Migliori and coworkers [32]. These Authors compared normal rats, nephrectomized rats and nephrectomized rats treated with Fluoxetin, and found a significant decrease in brain derived neurotrophic factor (BDNF) at the level of the prefrontal cortex in the nephrectomized rats compared to normal rats. Moreover, they showed a partial recovery in the Nx-F rats [32]. The reduced BDNF had been widely related to atrophy and cellular death of glia and neurons in neurodegenerative disorder [32]. Previous PET studies also revealed abnormalities in these areas. In an F-18-fluorodeoxyglucose (FDG) PET study, Song et al. found several voxel clusters of significantly decreased cerebral glucose metabolism in pre-dialysis CKD patients, including the left prefrontal cortex (Brodmann's area 9), the right prefrontal cortex (Brodmann's area 10) and the right basolateral prefrontal cortex (Brodmann's area 46), the left anterior cingulate gyrus (Brodmann's area 32), the left premotor cortex (Brodmann's area 6), the left transverse temporal gyrus (Brodmann's area 41), the left superior temporal gyrus (Brodmann's area 42), the right basolateral prefrontal cortex (Brodmann's area 44), the right inferior parietal lobule (Brodmann's area 39), the left middle temporal gyrus (Brodmann's area 19), and the left angular gyrus (Brodmann's area 39). Moreover, they found a negative correlation between the cerebral glucose metabolism of the right orbitofrontal cortex and the Hamilton Depression Rating Scale (HDRS) in pre-dialysis CKD patients (Brodmann's area 11) [12]. Through measuring brain oxygen metabolism, Kanai et al. demonstrated significantly lower values of hemispheric and cerebral cortex oxygen metabolism in both hemodialysis and CKD patients compared with controls, and the frontal cortices tended to show the lowest values in patients with renal failure [11].

However, our VBM findings were somewhat different from previous VBM studies. Through comparing minimal nephro-encephalopathy (MNE) and Non-MNE, with controls, Zhang et al. reported diffusely decreased gray matter volumes in ESRD patients. In addition, they found that serum urea was negatively associated with changes in gray matter volume in many regions (bilateral occipital lobes, bilateral lingual lobes, bilateral calcarine, bilateral superior temporal gyri, bilateral temporal poles, bilateral uncus, posterior cingulate cortex/precuneus/cuneus, right fusiform, right parahippocampus, right amygdala, left hippocampus/parahippocampus) [24]. Thus, the differences in the laboratory tests (especially for the serum urea) might be one of the potential reasons. Another possible mechanism might be the hemodialysis differences in the ESRD patients between the two studies. In contrast to our present study, in which all the ESRD patients were undergoing hemodialysis, only 33 of 57 ESRD patients were on hemodialysis in their study [24]. Studies based on transcranial Doppler have indicated a decrease in the mean flow velocity (mfv) at the level of the middle cerebral artery (MCA) during hemodialysis, MCAmfv has been proposed as a reliable proxy for cerebral blood flow [33]–[35]. In addiction, lower cerebral blood flow has always been associated with lower brain gray matter volume and lower cortical thickness [36]–[38]. In a study by Prohovnik et al., 10 ESRD patients on hemodialysis and 6 controls were compared, and they found decreased gray volume only in bilateral caudate nuclei but not in other regions [25]. The most likely cause for the difference from our study may be the sample size.

The dorsal lateral prefrontal cortex serves as the highest cortical area responsible for motor planning, organization, and regulation [39]. OFC is involved in cognitive processing of decision-making [40]. Damage to either of these regions can result in the dysexecutive syndrome [41], which leads to problems with emotion, social judgment, executive memory, abstract thinking and intentionality. The decreased gray matter volume in these regions observed in the present study may imply executive function deficits in ESRD patients, which is supported by previous neuropsychological studies indicating that executive function deficits were the prominent feature of cognitive impairment among ESRD patients [42].

How the gray matter structural abnormalities in ESRD patients relate to cerebral functional integrity deficits is an interesting question. In the present study, regions with abnormal gray matter volume were used as seeds for functional connectivity analysis. We found a disconnect between the prefrontal cortex and the bilateral cerebellum (consist fronto-cerebellar circuits), and within the prefrontal cortex. We also found enhanced functional connectivity between the prefrontal cortex and left middle temporal gyrus as well as within the prefrontal cortex in ESRD patients when compared to the healthy controls (Figure 2, Table 3). Moreover, supplementary analysis showed that most of the results remained significant when local gray matter volumes (except the left dorsal lateral prefrontal cortex) were statistically controlled for. This suggests that local gray matter volumes partially influenced the functional results, but that the abnormalities we found regarding resting state functional connectivity in the ESRD group cannot entirely be explained by their lower gray matter volume.

Fronta-frontal circuits including the dorsolateral circuit, orbitofrontal circuit and anterior cingulate cortex circuit are thought to be involved in attention, cognition, action and emotion [43]. The separation performances of functional connectivity (enhance and reduce) within fronta-frontal circuits may represent different neural mechanisms, while reduced functional connectivity within the fronta-frontal circuits implies that the ESRD-related functional impairment, and enhanced functional connectivity may indicate compensatory mechanisms. Ideally, task-fMRI studies combined quantitative MRI imaging with neuropsychological testing should be planned to prove this hypothesis.

Fronto-cerebellar circuits include three distinct circuits that associate with the prefrontal cortex. These fronto-cerebellar circuits are thought to be involved in higher-order cognitive functioning. Studies have consistently demonstrated that the fronto-cerebellar circuits are associated with cognitive function [44], [45]. Disconnection of the fronto-cerebellar connectivity observed in the present study may contribute to cognitive deficits in ESRD patients. This hypothesis can be partly supported by previous studies on alcoholism, which indicated that the disconnection between the fronto-cerebeller circuits related to the cognitive deficits in alcoholics and alcohol-naïve youth with a family history of alcoholism [44], [45]. Further support to this hypothesis is also provided by a recent study performed in children with attention-deficit/hyperactivity disorder (ADHD), which found that the frontal and cerebellar circuits neural activity was enhanced in ADHD patients after cognitive training [46]. If this hypothesis holds, cognitive training can be used to enhance fronto-cerebellar connectivity of ESRD patients, which may improve the cognitive function in ESRD patients.

We also observed an enhanced FC between the left middle temporal gyrus and the medial PFC (Brodmann's area 10), while the fronto-temporal circuits function in language processing. The enhanced FC in this circuit may be compensatory for the GM volume reduction in the left temporal gyrus.

We did not find any correlation between the brain deficits (structural and functional) and clinical parameters in ESRD patients. Several factors might explain these findings. First, depression is the most common psychological disorder in ESRD patients with a prevalence as high as 20–25% by some contemporary estimates [47]. The reduced gray matter volume in the bilateral OFC, the left middle temporal gyrus and the bilateral dlPFC observed in present study is also found in patients with depression [48]. Thus, the brain deficits observed in the present study may result from the complication (depression) and not from the ESRD itself. A more rigorous experiment to exclude the effects of depression is needed in the future. Second, a relatively small sample size may lead to insufficient power. Although we did not find any significant correlation between the brain deficits and clinical parameters in ESRD patients, we found negative trends between the serum urea levels and the bilateral OFC, the left middle temporal gyrus and the right dlPFC gray matter volume. A larger sample size is needed in future studies.

### Limitations

We acknowledge that our study has some limitations. The main limitation of the study is that all of the ESRD patients received regular hemodialysis at the time of the fMRI study. Whether and how hemodialysis itself can affect the brain is unknown; however, it can affect the patient's cognitive function [49], [50]. Although we did not find any significant correlation between the abnormal gray matter volume, FC and times of hemodialysis, a more detailed experiment with chronic kidney disease (stage 4–5) without hemodialysis is required in the future study. Second, although we temporally bandpass filtered all fMRI data (0.01–0.08 Hz), and removed components with high correlation to cerebrospinal fluid or white matter or with low correlation to gray matter, we cannot completely rule out the influence of physiological noise on our findings due to its variation over time and across subjects. Simultaneous recording of heart rate and respiratory rate and depth during fMRI scanning might help further reduce physiological noise artifacts. Another limitation is that the current study did not include cognitive testing to allow the examination of any correlation with the structural brain abnormalities and functional connectivity. Such an investigation might potentially improve our understanding of the pathophysiological mechanisms of ESRD. In addition, this study is preliminary and our results are limited to a small sample size, which may affect the statistical analysis. Further studies with large-cohort are needed.

## Conclusions

In conclusion, the present study applied morphometry analysis and resting-state functional connectivity to examine the structural and functional integrity changes in ESRD patients. Our findings document that patients with ESRD undergoing routine hemodialysis display clear-cut structural alterations in selected gray matter areas, Moreover, regions with gray matter volume reduction have significantly altered resting state functional connectivity with other brain regions. Our results provide support for future efforts to combine anatomical and functional data to explore the cognitive deficits of ESRD patients.
